# Retentive force of thermoformed and direct 3D-printed clear aligners with different margin designs and attachments: an *in vitro* study

**DOI:** 10.3389/froh.2026.1881161

**Published:** 2026-07-08

**Authors:** Thi Bich Van Tran, Thi Nha Ca Pham

**Affiliations:** Faculty of Dentistry, University of Medicine and Pharmacy at Ho Chi Minh City, Ho Chi Minh City, Vietnam

**Keywords:** attachment, clear aligner retention, direct 3D-printed aligner, margin design, thermoformed aligner

## Abstract

**Introduction:**

Clear aligner retention is essential for maintaining appliance stability, intimate aligner fit, and effective orthodontic force delivery. With the increasing interest in direct 3D-printed aligners (DPA), comparative evidence on their retention behavior relative to conventional thermoformed aligners (TFA) under different design conditions remains limited.

**Objective:**

This *in vitro* study aimed to compare the retentive force of TFA and DPA under different margin designs and attachment conditions.

**Methods:**

A total of 72 aligners were fabricated using two Methods: thermoforming with Zendura VIVA, 0.89 mm, and direct 3D printing with Tera Harz TC-85DAC, designed thickness 0.50 mm. Specimens were assigned to 12 groups according to fabrication method, attachment condition (NA or YA), and margin design: Straight 2 mm, Straight 0 mm, or Scalloped 0 mm. Retentive force was measured using a universal testing machine at a crosshead speed of 5 mm/min. Each aligner was tested five times. Group comparisons were performed using the Mann–Whitney *U*-test and Kruskal–Wallis test, with significance set at *p* < 0.05.

**Results:**

TFA generally demonstrated higher retentive force than DPA, but lower retentive force in the Scalloped 0 mm design without attachments. The Straight 2 mm design produced the highest retentive force across groups, whereas the Scalloped 0 mm design showed the lowest values. The highest mean retentive force was observed in the TFA-YA Straight 2 mm group (26.18 ± 1.67 N), and the lowest in the TFA-NA Scalloped 0 mm group (3.25 ± 0.94 N). Attachments increased retentive force in most conditions, with a pronounced effect in the Scalloped 0 mm design.

**Conclusion:**

Clear aligner retention varied according to fabrication method, margin design, attachment condition, and material-related characteristics. Individualized aligner design may help optimize retention and improve biomechanical performance in clear aligner therapy.

## Introduction

Clear aligner therapy (CAT) has been increasingly adopted in orthodontic treatment because of its esthetics, removability, and patient comfort (1). As digital orthodontics continues to evolve, direct 3D-printed aligners (DPA) are emerging as a promising approach with the potential to reshape clear aligner manufacturing. Unlike conventional thermoformed aligners (TFA), which require an intermediate dental model for each treatment stage, DPA can be fabricated directly from a digital design. This model-free workflow may shorten production time, reduce cumulative manufacturing errors, and decrease material consumption and plastic waste associated with the mass production of printed dental models (2, 3). From this perspective, proponents of DPA suggest that eliminating the intermediate model and thermoforming steps may improve aligner adaptation, enhance retentive performance, and require fewer or no conventional attachments, which can be bulky, compromise esthetics, and reduce patient comfort, particularly in the anterior region. Beyond manufacturing efficiency, DPA offers new opportunities for individualized aligner design. Direct 3D printing allows local control of aligner thickness, internal geometry, and pressure-generating areas at the tooth level (4). These design capabilities may enable more selective force delivery and improve control of prescribed tooth movements (5).

The development of shape-memory polymers has further increased interest in DPA. These materials can be thermally activated and recover toward their original shape under conditions close to the intraoral environment, potentially improving adaptation to tooth morphology and maintaining more stable orthodontic forces over time. The elastic, viscoelastic, and shape-memory properties of these materials may support individualized biomechanical control by improving aligner adaptation and force delivery (6, 7). Despite these potential advantages, DPA also presents important technical and biological challenges. Direct printing requires specialized equipment, validated printing parameters, and strict post-processing protocols to ensure dimensional accuracy and reduce the risk of residual uncured resin and cytotoxicity (8). Moreover, although DPA offers theoretical advantages in fit, force control, environmental sustainability, and attachment reduction, its retention behavior compared with conventional TFA remains insufficiently understood.

Stable retention is essential for effective orthodontic tooth movement with clear aligners. Adequate retention maintains intimate aligner fit, supports force transmission, and resists dislodging forces generated by tongue activity, gravity, aligner insertion and removal, and interarch elastics (9). Aligner retention is influenced by multiple factors, including material properties, thickness, fabrication method, attachment design, and margin design (10–12). Among design-related factors, margin design is clinically important because it affects both aligner retention and patient comfort. Scalloped margins may provide better esthetics and soft-tissue comfort but generally show lower retention, whereas extended straight margins may increase retention by increasing cervical coverage and contact area. Straight margins at the gingival margin may represent an intermediate design that balances retention and comfort (11–13). However, direct comparative evidence on how different margin designs influence the retention of TFA and DPA, with or without attachments, remains limited.

Therefore, this *in vitro* study aimed to compare the retentive force of TFA and DPA under different margin designs and attachment conditions. The findings may provide a scientific basis for optimizing aligner design and selecting appropriate fabrication methods to improve the biomechanical performance of CAT.

## Materials and methods

### Study design

**Figure 1:**
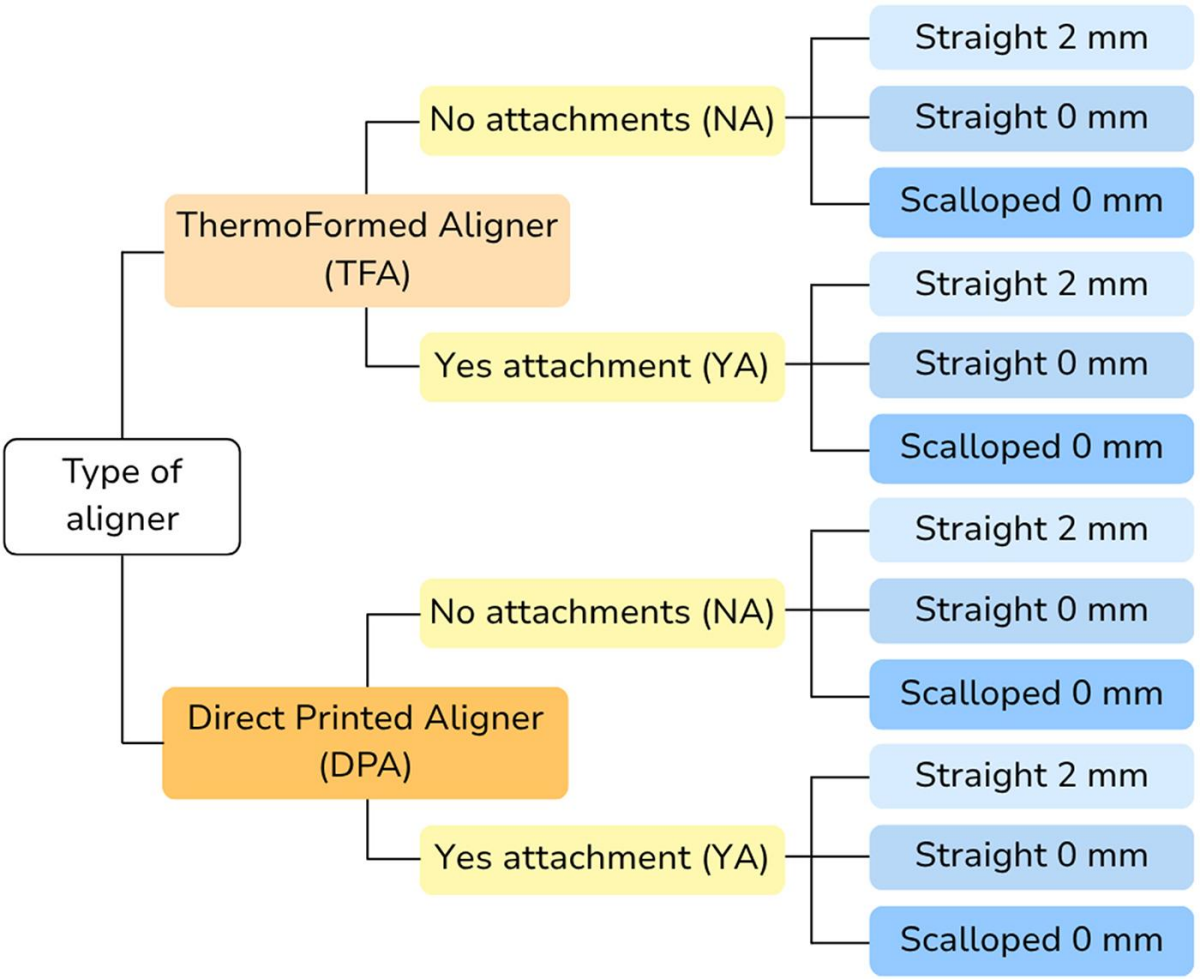
Aligner grouping by fabrication, attachment, and margin design.

An *in vitro* experimental study was conducted to evaluate the effects of three independent variables on the retentive force of clear aligners (CAs). A total of 12 experimental groups were defined based on combinations of two fabrication methods, direct 3D-printed aligners (DPA) and thermoformed aligners (TFA); two attachment conditions, with four attachments (YA) and without attachments (NA); and three margin designs: straight margin extending 2 mm above the gingival margin (Straight 2 mm), straight margin at the gingival margin (Straight 0 mm), and scalloped margin at the gingival margin (Scalloped 0 mm) (Figure 1).

### Sample size calculation

Based on previous *in vitro* studies (11, 12) by Cowley et al., 2012 and Cremonini et al., 2025 and considering laboratory feasibility, six specimens were included in each experimental group. Accordingly, a total of 72 aligners were fabricated. Each specimen was tested five times, and the mean value of the repeated measurements was used for statistical analysis.

The statistical power of the significant results obtained was calculated using an a posteriori (post-hoc) power analysis with G*Power software, version 3.1.9.7 (Heinrich-Heine-Universität Düsseldorf, Düsseldorf, Germany). For comparisons among the three margin designs within each fabrication and attachment subgroup, the closest G*Power framework for the Kruskal–Wallis test was “ANOVA: fixed effects, omnibus, one-way”, with *α* = 0.05, total sample size = 18, and number of groups = 3; the achieved power was greater than 0.99. For significant pairwise comparisons between two independent groups, including comparisons between fabrication methods and attachment conditions, the closest framework for the Mann–Whitney *U*-test was “Means: difference between two independent means (two groups)”, with a two-tailed test, *α* = 0.05, n1 = 6, and n2 = 6. The achieved power ranged from 0.89 to greater than 0.99, all exceeding 80%.

### Materials

Two advanced materials representing contemporary aligner fabrication technologies were investigated: Zendura VIVA (ZV), a multilayer thermoplastic polymer used for thermoforming, and Tera Harz TC-85DAC (TC-85), a shape-memory photopolymer resin used for direct 3D printing. Detailed material characteristics are summarized in Table 1.

**Table 1:** Characteristics of the investigated aligner materials (manufacturers' Data).

**Parameter**	**Zendura VIVA (ZV)**	**Tera Harz TC-85DAC (TC-85)**
Manufacturer	Bay Materials LLC, Fremont, CA, USA	Graphy Inc., Seoul, South Korea
Thickness (mm)	0.89 mm	Designed thickness: 0.50 mm
Lot number	250 2–37	1-EB11B01098
Fabrication method	Thermoforming: Heating for 50 s at 220°C, and pressure-forming at 4.8 bar, then cooling for 60 s	3D printing using a DLP-type 3D printer (Uniz UBEE; Uniz, San Diego, CA, USA) at a layer thickness of 100 μm, followed by UV light post-curing at 405 nm under N₂ conditions for 25 min using a post-curing device (Tera Harz Cure; Graphy, Seoul, South Korea)
Material composition	Three-layer sheet of a middle thermoplastic soft elastomeric TPU layer and two hard layers of co-polyester	Urethane acrylate oligomer with acrylic monomers
Regulatory certification	Class I medical device (EU Medical Device Directive 93/42/EEC)	CE-, FDA-, and KFDA-certified material

### Sample preparation

**Figure 2:**
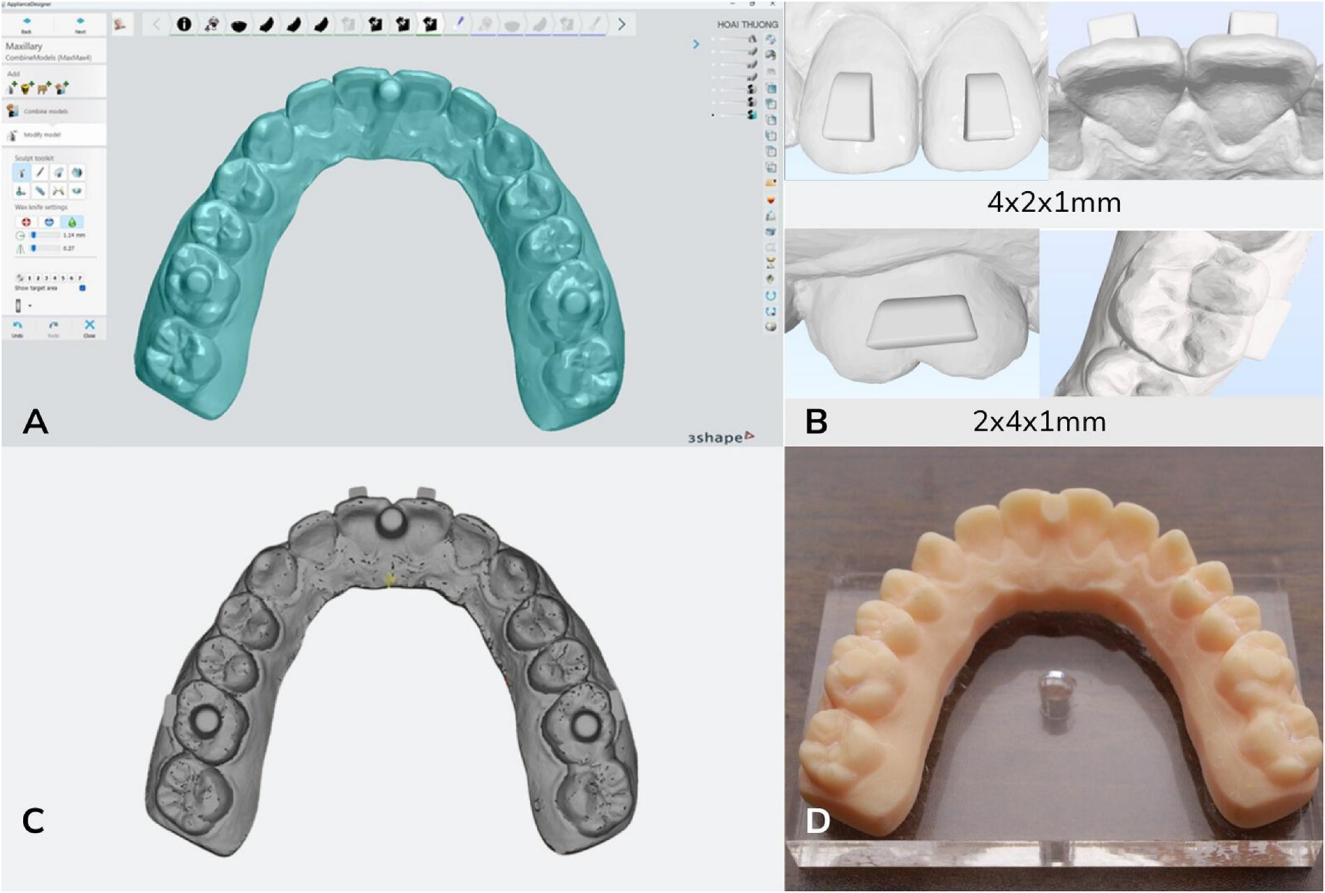
Model design and fabrication. **(A,C)** Digital maxillary models (NA and YA) created in Appliance Designer software with blocking structures positioned on occlusal and palatal surfaces. **(B)** Attachment design, including vertically and horizontally oriented rectangular attachments with gingivally beveled surfaces. **(D)** Final 3D-printed model mounted on the base plate for experimental testing.

A master scan of a maxillary arch was obtained using a TRIOS 3 (3Shape, Copenhagen, Denmark) intraoral scanner and processed in Appliance Designer software (3Shape, Copenhagen, Denmark). Two digital models were generated: one without attachments (NA) and one with attachments (YA). In both models, blocking structures were designed as cylindrical units (3 mm in diameter and 3 mm in height) and positioned on the occlusal surfaces of teeth 16 and 26, as well as on the palatal surface between teeth 11 and 21 (Figures 2A,C).

In the YA model, attachments were placed on the buccal surfaces. Vertically oriented rectangular attachments with a gingivally beveled surface (4 mm in height, 2 mm in width, and 1 mm in depth) were positioned on teeth 11 and 21, while horizontally oriented rectangular attachments with a gingivally beveled surface (2 mm in height, 4 mm in width, and 1 mm in depth) were positioned on teeth 16 and 26 (Figure 2B).

A total of 72 models (36 NA and 36 YA) were fabricated using a 3D printer, Rayshape Edge E1 (Suzhou, China), with photopolymer resin, Jamghe ECO Standard (China). Post-processing included ultrasonic cleaning in 95% isopropyl alcohol for 5 min, followed by UV curing for 15 min using a curing unit, Elegoo Mercury X Wash (China). A custom acrylic base plate (7 × 5 × 0.8 cm) with a central hole (5 mm in diameter) was used to secure the models during testing (Figure 2D).

Separate corresponding models were used for retentive force measurements in the TFA and DPA groups. All testing models were generated from the same original digital master model and the same NA and YA configurations to standardize model geometry and dimensions across groups. Each aligner was tested on its corresponding coded 3D-printed model.

### Fabrication of TFA and DPA

A total of 72 aligners were fabricated and divided into two groups according to fabrication method: TFA (*n* = 36) and DPA (*n* = 36).

**Figure 3:**
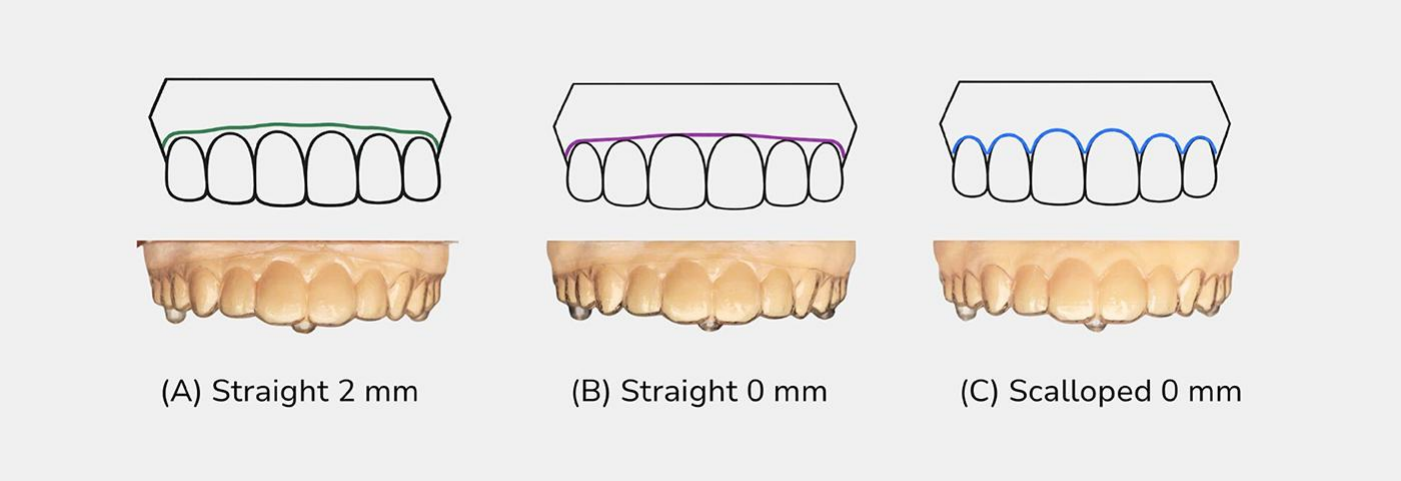
Gingival-Margin designs of aligners tested in this study.

For the TFA group, Zendura VIVA (ZV) thermoplastic sheets (nominal thickness: 0.89 mm) were thermoformed over 36 3D-printed models using a pressure-forming machine (Drufomat, Scheu-Dental, Germany). The thermoforming process was performed according to the manufacturer's recommended parameters (Table 1) to standardize fabrication. During heating, a horizontal laser beam was projected parallel to the thermoplastic sheet within the heating chamber and used as a fixed reference plane. The vertical deflection of the sheet, defined as the sag depth, was measured relative to this laser reference plane. Heating was terminated when the central deflection of the sheet reached 16 mm relative to the laser plane. The aligners were then trimmed and polished according to the three margin designs: Straight 2 mm, Straight 0 mm, and Scalloped 0 mm (Figure 3), by an experienced technician. A total of 36 thermoformed aligners were produced, including 18 without attachments (TFA-NA) and 18 with attachments (TFA-YA). For each TFA specimen, the same corresponding 3D-printed model used for thermoforming was subsequently used for retentive force measurement. This approach ensured geometric consistency between fabrication and testing.

For the DPA group, 36 aligners were digitally designed using Digital Aligner Design software (DAD, Graphy, Seoul, Korea) with a uniform thickness of 0.50 mm and an offset of 0.05 mm. Two configurations were created: without attachments (DPA-NA) and with attachments (DPA-YA), each incorporating the three margin designs described above. The designs were exported as STL files, imported into printing software (Uniz, San Diego, CA, USA), oriented at 30°, and supported with support structures.

The aligners were printed using a 3D printer (Uniz UBEE, CA, USA) with a layer thickness of 100 µm, using photopolymer resin (Graphy Tera Harz TC-85DAC, Graphy Inc., Seoul, Korea). Post-processing followed the Tera Harz system protocol, including centrifugal cleaning for 3 min to remove uncured resin, removal of supports, and post-curing using a curing unit (Tera Harz Cure, Graphy Inc., Seoul, Korea) according to the manufacturer's specifications (Table 1). The aligners were then polished by an experienced technician. A total of 36 direct 3D-printed aligners were fabricated, including 18 DPA-NA and 18 DPA-YA. All 72 aligners were evaluated for fit on their corresponding models and randomly coded prior to retentive force testing. TFA and DPA specimens were fabricated in parallel during the same preparation phase. After finishing, fit verification, and coding, all aligners and their corresponding models were stored at room temperature under standard laboratory conditions in separate containers until testing to avoid deformation or mechanical damage. During the testing phase, retentive force measurements were performed sequentially, and specimens awaiting testing were maintained under the same storage conditions.

### Measurement of retentive force

Retentive force was defined as the peak tensile force required to dislodge the aligner from the dental model and was recorded in Newtons (N) using a Materials & Structures Testing Machine PNXUCT (Hoang Vinh T.R.C.C Ltd, Vietnam). The system was equipped with a load cell CSBA-50L, 50 kg capacity (Curiotec, Korea) and calibrated prior to testing using a digital force gauge SHAHE AFM-500 (SHAHE, China).

**Figure 4:**
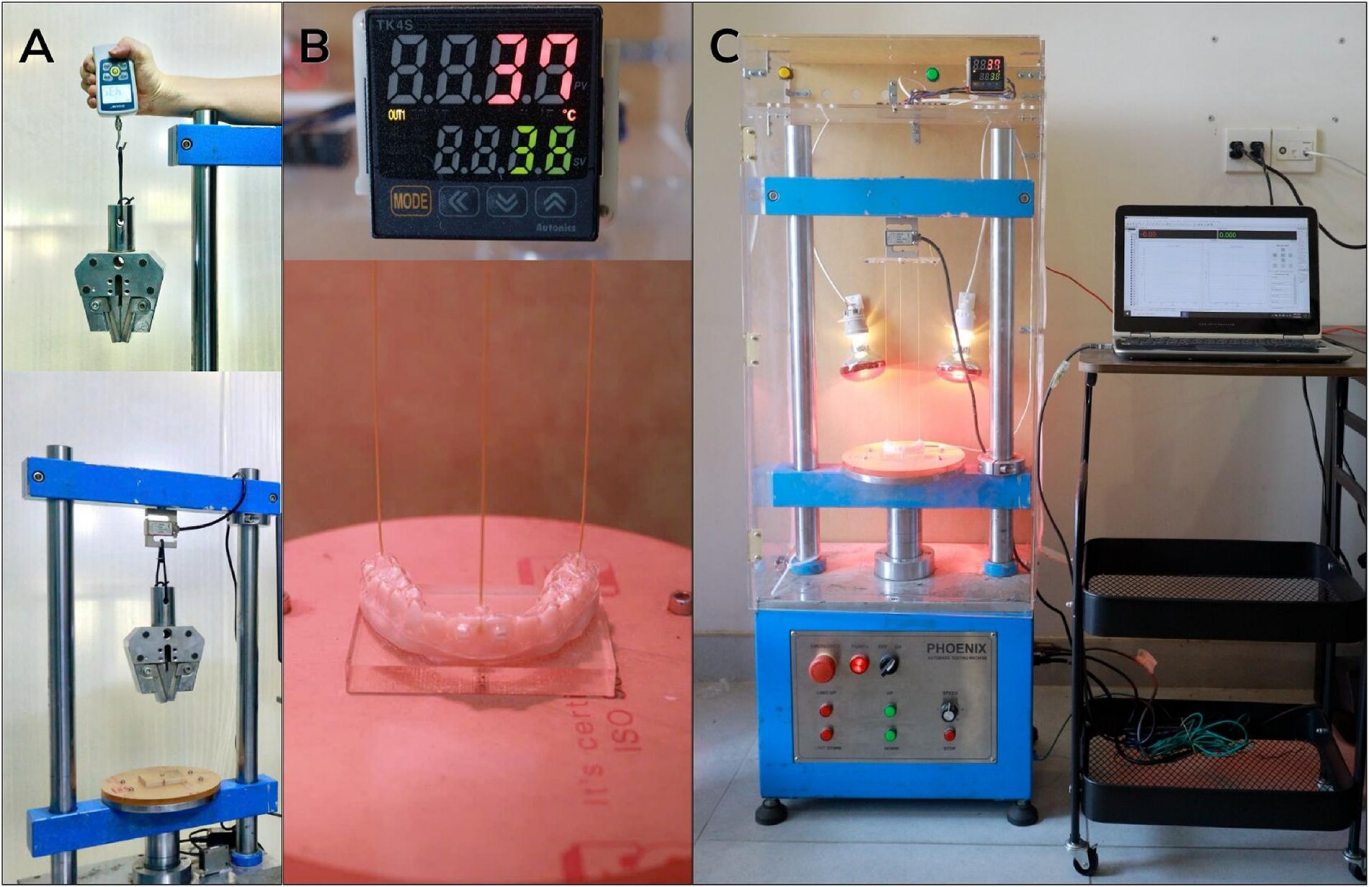
Experimental setup for measuring aligner retentive force. **(A)** Calibration of the force system prior to testing; **(B)** Stabilization of the dental model; **(C)** Force measurement and data recording.

Before testing, the blocking cylinders on the models were removed, and a 1-mm hole was created in each aligner to attach the pulling wire. The model was securely fixed to the testing platform. Each aligner was fully seated on the model, and the pulling wire was connected to the load cell, ensuring a vertical dislodging direction perpendicular to the occlusal plane (Figure 4).

For DPA specimens, the aligners were thermally activated by immersion in water at 80°C for 30 s and then seated onto the model with gentle pressure to ensure full adaptation. This activation step was applied only to DPA specimens because TC-85 is a temperature-sensitive shape-memory resin, whereas TFA specimens did not require thermal activation. After seating, DPA specimens were maintained at 37°C for 1 h to allow shape recovery before testing. TC-85 has a cross-linked polymer network that enables shape recovery. Previous data showed that approximately 90% of deformation recovered within 10 min, and the recovery ratio reached 96% after 60 min, indicating excellent shape-memory properties (6).

After confirming the initial seating, the force measurement system was zeroed. A tensile force was applied at a constant crosshead speed of 5 mm/min until the aligner was completely removed from the model. The peak tensile force recorded immediately before complete dislodgement was defined as the retentive force. The aligner was then reseated on its corresponding coded model, and the procedure was repeated for subsequent measurements. All measurements for both TFA and DPA specimens were performed at 37°C to simulate intraoral conditions. Each specimen was tested five times, and the mean value was used for statistical analysis.

### Statistical analysis

All data were recorded in Microsoft Excel and analyzed using Stata MP 17.0 (StataCorp, College Station, TX, USA). The mean retentive force from five repeated measurements of each aligner was used as the unit of analysis. Numerical data were presented as mean ± standard deviation (SD). The Shapiro–Wilk test was used to assess the normality of data distribution. Because the retentive force data were not normally distributed, non-parametric tests were applied. The Mann–Whitney *U*-test was used for pairwise comparisons between two independent groups, including comparisons between fabrication methods and attachment conditions. The Kruskal–Wallis test was used to compare retentive forces among the three margin designs within each fabrication method and attachment condition, followed by Dunn's *post hoc* test with Bonferroni correction when significant differences were detected. Statistical significance was set at *p* < 0.05.

## Results

### Comparison of retentive force between TFA and DPA

**Figure 5:**
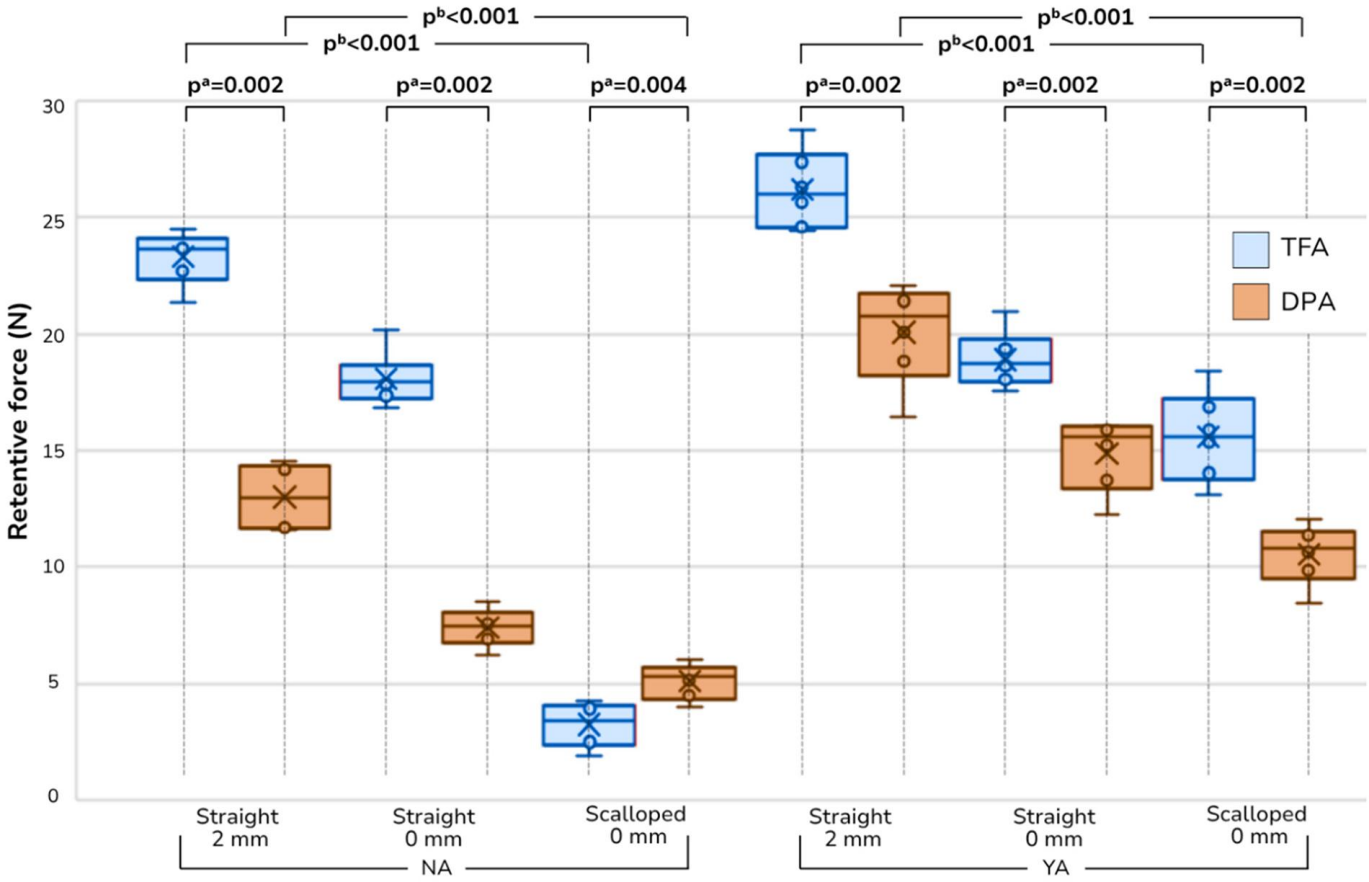
Comparison of retentive force between fabrication methods. *p*ᵃ: Mann–Whitney *U*-test; *p*ᵇ: Kruskal–Wallis test.

As shown in Figure 5, in the NA group, TFA showed significantly higher retentive force than DPA in the Straight 2 mm and Straight 0 mm designs (both *p*ᵃ = 0.002). Conversely, DPA showed significantly higher retentive force than TFA in the Scalloped 0 mm design (*p*ᵃ = 0.004).

In the YA group, TFA demonstrated significantly higher retentive force than DPA across all three margin designs (all *p*ᵃ = 0.002). Within both TFA and DPA, retentive force differed significantly among the three margin designs in both the NA and YA groups (all *p*ᵇ < 0.001).

### Comparison of retentive force among margin designs

**Figure 6:**
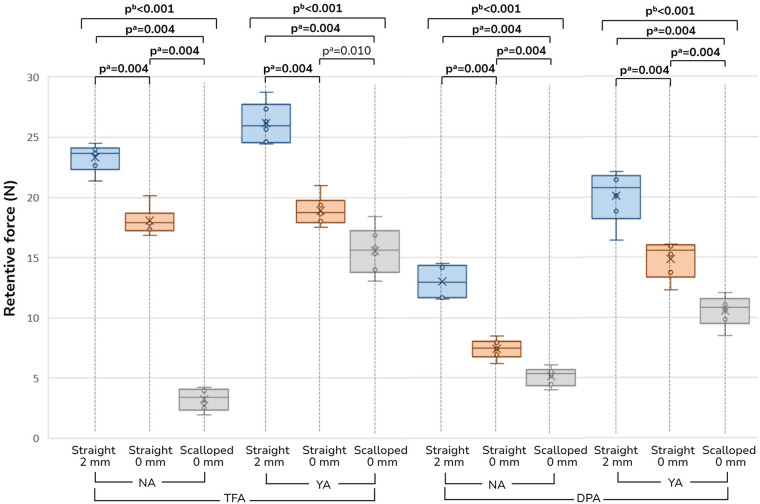
Comparison of retentive force among margin designs. *p*ᵃ: Dunn's *post hoc* test with Bonferroni correction; *p*ᵇ: Kruskal–Wallis test.

According to Figure 6, margin design significantly influenced retentive force across all fabrication and attachment conditions (all *p*ᵇ < 0.001). Across all groups, the Straight 2 mm design produced the highest retentive force, whereas the Scalloped 0 mm design showed the lowest values. The highest mean retentive force was observed in the TFA-YA Straight 2 mm group (26.18 ± 1.67 N), while the lowest value was found in the TFA-NA Scalloped 0 mm group (3.25 ± 0.94 N). Pairwise comparisons confirmed significant differences among all three margin designs in all conditions (all *p*ᵃ ≤ 0.010).

### Comparison of retentive force by attachment condition

**Figure 7:**
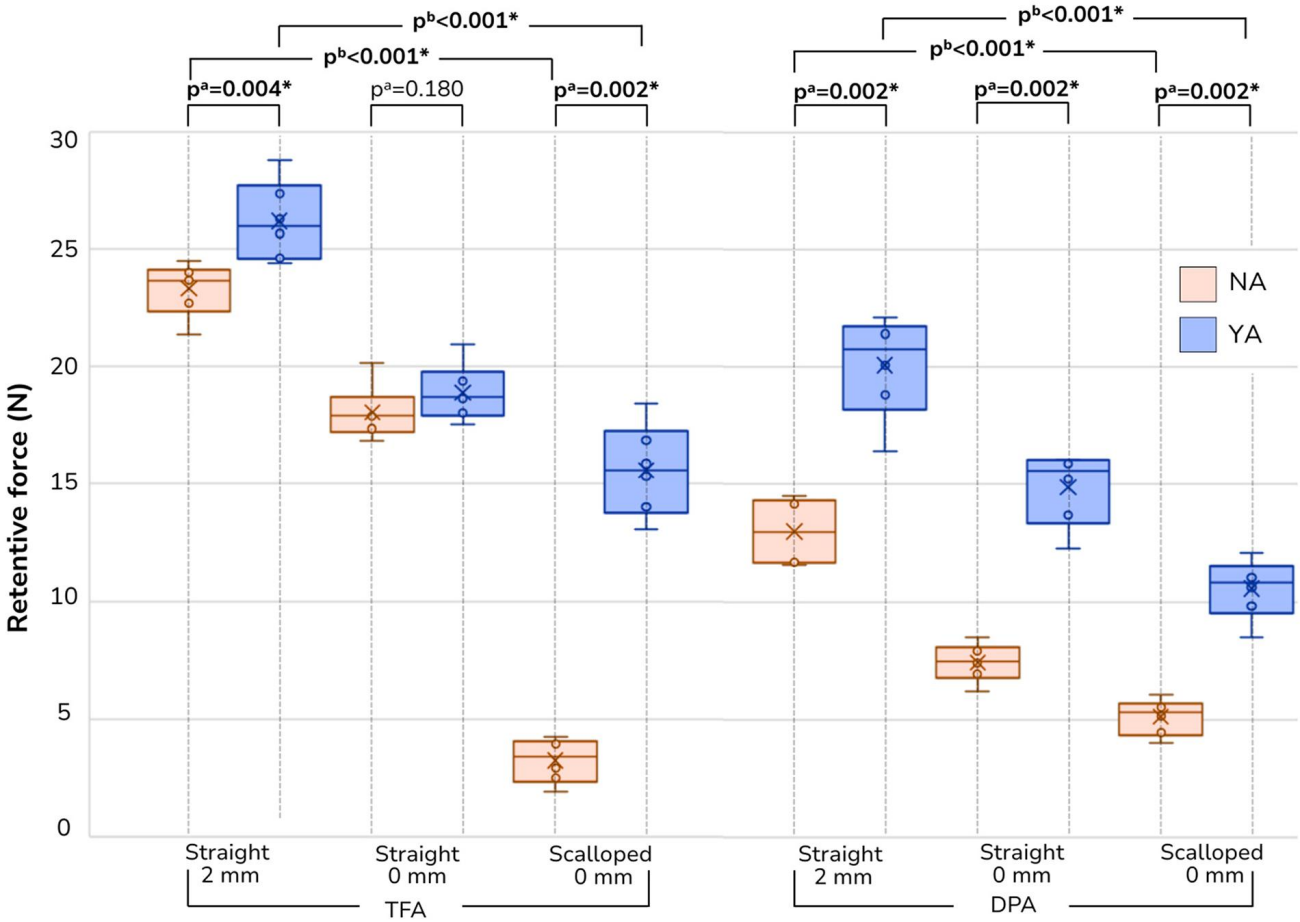
Comparison of retentive force by attachment condition. *p*ᵃ: Mann–Whitney *U*-test; *p*ᵇ: Kruskal–Wallis test.

As shown in Figure 7, YA showed significantly higher retentive force than NA in most groups. In TFA, YA showed significantly higher retentive force than NA in the Straight 2 mm and Scalloped 0 mm designs (*p*ᵃ = 0.004 and *p*ᵃ = 0.002, respectively), whereas no significant difference was observed in the Straight 0 mm design (*p*ᵃ = 0.180). In DPA, YA demonstrated significantly higher retentive force than NA across all three margin designs (all *p*ᵃ = 0.002). In the Scalloped 0 mm design, retentive force increased from 3.25 ± 0.94 N to 15.59 ± 1.93 N in TFA and from 5.13 ± 0.76 N to 10.56 ± 1.26 N in DPA after attachment placement.

## Discussion

The present study demonstrated that clear aligner retention varied according to fabrication method, margin design, and attachment condition. Differences in retentive force between TFA and DPA depended on margin design and attachment condition, suggesting that retention should be interpreted within the combined context of material behavior and aligner geometry. Among the three margin designs, Straight 2 mm consistently produced the highest retentive force, whereas Scalloped 0 mm produced the lowest. Attachments increased retentive force in most conditions, with the most pronounced effect observed in the Scalloped 0 mm design. These findings indicate that aligner retention is not determined by a single factor, but by the combined effects of fabrication method, material behavior, margin height, and attachment configuration.

When comparing TFA and DPA, TFA generally produced higher retentive force across most margin designs, particularly in the YA groups. However, in the NA group with the Scalloped 0 mm design, DPA showed significantly higher retentive force than TFA. This finding suggests that the difference between the two aligner types cannot be attributed solely to material composition, but should be interpreted within the context of the entire material-fabrication system, including material properties, manufacturing process, aligner fit, and post-fabrication thickness.

Material behavior may partly explain these differences. TFA is fabricated from relatively stiff thermoplastic sheets; therefore, its retention may depend mainly on structural rigidity, margin extension, and attachment engagement. Rather than using Zendura FLX, which is more commonly used for routine aligner treatment, the present study tested Zendura VIVA in the TFA group. Zendura VIVA is a thicker material intended for selected cases or movements requiring enhanced force delivery. Because material selection is an important factor influencing aligner retention, these findings provide a preliminary basis for future studies using additional thermoformed materials with different elastic and thermoplastic properties, such as PET-G (Polyethylene Terephthalate Glycol) or commercially available polyurethane-based materials, to further evaluate how material type interacts with aligner design factors. By contrast, DPA is directly fabricated from photopolymerizable resin and may exhibit elastic deformation and shape-memory behavior, with thermal activation at high temperature and shape recovery at 37°C, approximating the intraoral environment (1, 2). These properties may enhance intimate adaptation between the inner aligner surface and tooth surface, engagement with cervical areas and undercuts, and elastic recovery after seating. In low-retention situations, such as the Scalloped 0 mm design without attachments, these material- and fit-related characteristics may partly explain why DPA showed higher retentive force than TFA.

Aligner fit and post-fabrication thickness should also be considered when interpreting the retention differences between TFA and DPA. The gap between the inner aligner surface and the tooth surface may affect orthodontic force transmission, retention, and comfort, because part of the generated force can be absorbed by this gap and by the elasticity of periodontal tissues (14). Previous studies have reported that DPA may show higher trueness and precision than corresponding thermoformed aligners, resulting in better fit (15–17). However, DPA fit can still be influenced by printer type, material, printing parameters, post-processing protocol, and tooth morphology. In addition, nominal material thickness may not reflect actual post-fabrication thickness. Because the actual thicknesses of TFA and DPA specimens were not directly measured or standardized in the present study, the comparison between fabrication methods should be interpreted within the context of their respective material-fabrication systems rather than as a thickness-controlled material comparison. Thermoformed sheets may undergo non-uniform thinning during thermoforming (6, 18), whereas DPA may become thicker than the designed value because of residual resin after printing and post-processing. Previous studies reported that DPA may be approximately 0.2 mm thicker than the designed thickness (4), or about 12% thicker than the digital design value (6). These deviations may influence aligner fit, stiffness, biomechanical behavior, and retentive force. Therefore, future studies should measure and, where possible, standardize TFA and DPA thickness before mechanical testing.

Margin design showed a consistent influence on retentive force across all tested conditions. The Straight 2 mm design produced the highest retentive force, followed by the Straight 0 mm design, whereas the Scalloped 0 mm design showed the lowest retentive force, regardless of fabrication method or attachment condition. This finding is consistent with previous studies showing that apically extended aligner margins increase retentive force compared with scalloped designs (12, 13, 19). Apical extension of the aligner margin increases cervical coverage and the contact area between the aligner and the tooth crown, thereby potentially improving retention, force transmission, and root movement control.

Attachments increased retentive force in most conditions, with a pronounced effect in the Scalloped 0 mm design. This finding is consistent with previous studies showing that attachments, particularly rectangular attachments, enhance aligner retention (10, 11). Clinically, when low-margin designs are selected for comfort, soft-tissue considerations, or esthetic reasons, attachments may be particularly important for improving mechanical retention and aligner stability, especially when cervical coverage and contact area are limited.

The findings of this study have biomechanical implications for clear aligner therapy. In particular, the highest mean retentive force was observed in the TFA-YA Straight 2 mm group (26.18 ± 1.67 N). Although this value indicates strong aligner retention, higher retentive force should not automatically be interpreted as ideal or clinically beneficial. Excessive retention may increase removal difficulty and patient discomfort, and may also cause fingernail or gingival damage, attachment debonding, or even unwanted luxation forces on the teeth during aligner removal. Clinically, when adequate retention has already been achieved, reducing the number or size of attachments, or selecting a less retentive margin design, may be considered to avoid over-retention. Previous *in vitro* studies have reported wide ranges of aligner removal or retentive forces depending on material, thickness, attachment design, margin configuration, and testing protocol (10–13). However, to the best of our knowledge, no universally accepted clinical threshold defining an ideal retentive force for clear aligners has been established. Future studies should define clinically acceptable ranges of aligner retention that balance appliance stability, biomechanical control, patient comfort, and ease of removal. Extended straight margin designs may improve retention and biomechanical control by increasing cervical coverage, enhancing force and moment transfer, and shifting the contact area closer to the tooth's center of resistance (19). These effects may be relevant for movements requiring better root control, such as translation, intrusion, en-masse retraction, maxillary arch expansion, and Class II elastic traction (20). However, the clinical relevance of margin extension may vary according to the intended tooth movement and patient-specific conditions. Extended margins may not be optimal in patients with pre-existing retentive factors, such as gingival recession, cervical abfraction, black triangles, or severe dental proclination, because they may increase discomfort during insertion and removal. By contrast, Scalloped 0 mm margins may be more suitable when comfort, esthetics, or reduced soft-tissue irritation is prioritized, although additional attachments may be required to ensure adequate retention.

Clinically, optimizing clear aligner retention requires a comprehensive biomechanical approach rather than reliance on a single design factor. Extended straight margins or attachments may be preferred when greater stability and force control are required, whereas lower margin designs or individualized DPA designs may be considered when esthetics, comfort, or reduced soft-tissue irritation is prioritized. The ability of DPA to incorporate region-specific thickness control allows local modification of aligner thickness, fit, and internal geometry directly at the digital design stage. This capability may represent a future direction for individualized aligner design, with the potential to improve selective force delivery and reduce reliance on conventional attachments.

## Conclusion

This *in vitro* study showed that the retentive force of clear aligners varied according to fabrication method, material characteristics, margin design, and attachment condition. TFA generally produced higher retentive force than DPA, although DPA showed higher retention in the Scalloped 0 mm design without attachments. Extended margins and attachments increased retentive force in most conditions, indicating that margin height and attachment configuration are important considerations for optimizing clear aligner retention. These findings may help guide individualized aligner design and fabrication choices according to clinical retention requirements.

### Limitations

Several limitations should be acknowledged. First, as an *in vitro* study, this experiment could not fully reproduce oral conditions, such as saliva, masticatory loading, temperature changes, material aging, and time-dependent aligner deformation. Second, retentive force was measured only in the vertical removal direction, which may not fully represent the multidirectional removal pattern in clinical practice. Third, aligner fit, internal gap, and actual post-fabrication thickness were not directly measured; therefore, interpretations related to adaptation, thickness, and retention should be considered with caution. From a long-term perspective, future studies incorporating water storage, thermal cycling, or repeated insertion-removal cycles may provide further insight into retention stability. Finally, the 80°C thermal activation protocol for DPA specimens was used as a standardized laboratory condition to reduce procedural variability before seating and testing. Although hot-water immersion is recommended for activating TC-85 aligners before use, patients may not always activate their aligners at the exact temperature and duration used in this experiment. Therefore, future studies should evaluate DPA retention under different patient-use and activation conditions.

## Data Availability

The original contributions presented in the study are included in the article/Supplementary Material, further inquiries can be directed to the corresponding author.
